# Vaccine hesitancy among primary care health workers in Campo Grande, after the COVID-19 pandemic

**DOI:** 10.1590/S2237-96222025v34e20240481.en

**Published:** 2025-06-20

**Authors:** Danilo dos Santos Conrado, Simony Portela do Carmo Drumond, Ana Isabel do Nascimento, Maria Elizabeth Araújo Ajalla, Cláudia Du Bocage Santos-Pinto, Everton Falcão de Oliveira

**Affiliations:** 1 Universidade Federal de Mato Grosso do Sul, Programa de Pós-Graduação em Doenças Infecciosas e Parasitárias, Campo Grande, MS, Brazil; 2 Universidade Federal de Mato Grosso do Sul, Faculdade de Medicina, Campo Grande, MS, Brazil

**Keywords:** Vaccine Hesitancy, Health Professionals, Primary Health Care, Misinformation, Cross-Sectional Studies, Vacilación a la Vacunación, Persona de Salud, Atención Primaria de Salud, Desinformación, Estudios Transversales

## Abstract

**Objective:**

To analyze vaccine hesitancy among primary care health workers, including the factors associated with it.

**Methods:**

Cross-sectional descriptive study carried out between November 2022 and August 2023 in the city of Campo Grande, Mato Grosso do Sul, through the application of a questionnaire on vaccine hesitancy proposed by the World Health Organization. The association of vaccine hesitancy with sociodemographic variables and workers’ perceptions was verified by bivariate analysis and multiple variables.

**Results:**

Vaccine hesitancy was observed in 32.7% of respondents, with a higher frequency after the start of the COVID-19 pandemic (64.9%). The coronavirus-19 vaccine had the highest frequency of hesitation (68.4%). Doctors and nurses were the least hesitant, while community workers were the most hesitant. Believing that there are reasons for people not to get vaccinated was associated with vaccine hesitancy (adjusted odds ratio (OR) 3.01; 95% confidence interval (95%CI) 1.60; 5.71). On the other hand, receiving institutional guidance to get vaccinated (OR 0.30; 95%CI 0.11; 0.78) and believing that hesitation affects the population’s vaccination coverage (OR 0.46; 95%CI 0.25; 0.83) were factors associated with low hesitation frequency.

**Conclusion:**

Vaccine hesitancy was common among primary care professionals. The COVID-19 pandemic and the infodemic that followed it appear to have contributed to this scenery. The need for interventions aimed at these workers is highlighted, in order to impact vaccination coverage of the general population.

## Resumen

**Objetivo:**

Analizar la vacilación a la vacunación entre los profesionales sanitarios de atención primaria, incluyendo los factores asociados a ella.

**Métodos:**

Estudio descriptivo transversal realizado entre noviembre de 2022 y agosto de 2023 en el municipio de Campo Grande, Mato Grosso do Sul, mediante la aplicación de un cuestionario sobre vacilación vaginal propuesto por la Organización Mundial de la Salud. Se verificó la asociación de la vacilación a la vacunación con variables sociodemográficas y percepciones de los trabajadores mediante análisis bivariado y de variables múltiples.

**Resultados:**

La vacilación a la vacunación se observó en el 32,7% de los dos entrevistados, con mayor frecuencia tras el inicio de la pandemia de covid-19 (64,9%). La vacuna contra el coronavirus-19 tiene la mayor frecuencia de dudas (68,4%). Los médicos y las enfermeras se muestran menos indecisos, mientras que los trabajadores comunitarios se muestran más indecisos. Creer que existen razones por las cuales las personas no se vacunan se asoció con la vacilación a la vacunación (*odds ratio*, OR ajustado) 3,01; intervalo de confianza (IC95%) (IC95% 1,60; 5,71). Por otro lado, recibir orientación institucional para vacunar (OR 0,30; IC95% 0,11; 0,78) y comprobar que la vacilación afecta la cobertura de vacunación de la población (OR 0,46; IC95% 0,25; 0,83) se asocian con una baja frecuencia de vacilación.

**Conclusión:**

La vacilación a la vacunación fue común entre los profesionales de atención primaria. La pandemia de Covid-19 y la infodemia que le siguió parecen haber contribuido a la situación. Destaca la necesidad de intervenciones dirigidas a estos trabajadores para incidir en la cobertura de vacunación de la población general.

**Ethical aspects**

This research respected ethical principles, having obtained the following approval data::

Research Ethics Committee: Universidade Federal de Mato Grosso do Sul

Opinion number: 5.200.726

Approval date: 1/13/2022

Certificate of Submission for Ethical Appraisal: 47947821.0.0000.0021

Informed Consent Form: Obtained from all participants prior to collection.

## Introduction

Vaccination is one of the main preventive and control measures for several infectious diseases, especially when high coverage percentages are achieved. However, vaccination uptake has declined worldwide, resulting in the reemergence of previously controlled diseases (1). 

One of the causes for the drop in vaccination coverage is vaccine hesitancy, defined as the delay, fear or even refusal to get vaccinated, regardless of the availability of vaccines in health services (2). To understand this phenomenon, in 2011 the World Health Organization (WHO) proposed a conceptual model, called 3C, which lists factors that determine vaccine hesitancy: trust, complacency and convenience (2). The model was expanded in 2018 to include the 5Cs, including calculus, which assesses the risks and benefits of vaccination, and collective responsibility, which refers to the willingness to protect other members of the community by vaccinating oneself (3).

In recent years, vaccine hesitancy has been intensified by the spread of fake news, fueled by content on digital media (4), contributing to what we call the “infodemic”. This refers to the excess of information about a certain health problem, associated with historical moments of insecurity, such as the recent COVID-19 pandemic (5).

Hesitation can be seen in the general population, but also among healthcare professionals. Studies indicate low vaccination adherence among these professionals in pre- and post-COVID-19 pandemic scenarios, even for vaccines already established in the National Immunization Program (PNI) routine (6,7). It is estimated that hesitant professionals, especially those working in primary health care (PHC), have an impact on the vaccination of their patients and, thus, on population coverage (4,8-11).

This study aims to analyze vaccine hesitancy among health workers in the PHC of Campo Grande, Mato Grosso do Sul, and the factors that determine it.

## Methods

### Study design and context

This is a cross-sectional study, carried out in Campo Grande, capital of Mato Grosso do Sul, between November 2022 and August 2023. At the time, there were 74 primary health care units in the municipality, 70 located in urban areas and 4 in rural areas, with 3,080 professionals linked to them (12). Of this total number of participants, it was not possible to quantify the occurrences of absences to work, such as those due to maternity leave and medical certificates, which may have affected the total number of professionals eligible for the study. Each unit was visited once by previously trained field researchers, after prior contact to schedule the visit. Workers were invited to participate and respond to the questionnaire at the time of the visit or, later, by accessing the link provided to complete the online questionnaire on the REDCap platform.

### Participants

All health workers over 18 years of age who were actively working in PHC at the time the research was conducted were considered eligible. 

### Variables

Vaccine hesitancy was defined as the dependent variable. Demographic, socioeconomic and behavioral data were used as independent variables. Reasons for hesitation reflected in professionals’ perceptions of vaccines (listed according to the 3C theoretical model), negative information about vaccines and their distribution periods, whether before or after the COVID-19 pandemic, were also investigated. Possible barriers to access and the influence of the work environment on the decision about vaccination were addressed.

### Data sources and measurement

The study was based on a questionnaire proposed by the WHO to assess vaccine hesitancy (13). Questions on demographic and socioeconomic characterization were included in this questionnaire. Participants were invited to respond and were initially presented with the WHO definition of vaccine hesitancy (2): “delay in accepting to be vaccinated or even refusing vaccination, regardless of the availability of vaccines in the SUS (Brazilian Unified Health System); hesitancy involves personal factors (such as religion, culture and politics), factors regarding trust in the health system and available vaccines (including fear of adverse effects) and the importance of vaccination and convenience of getting vaccinated (difficulty missing work, not seeing the need for vaccination, etc.)”.

 Based on this, the participants themselves answered whether they hesitated or not. When the participant responded that they were not hesitant in the first question “Considering the definition of vaccine hesitancy, have you ever hesitated to receive any vaccine?”, but in the following questions responded that they had refused any vaccine or had mentioned reasons or motives for refusing any vaccines, for analysis purposes, they were considered as hesitant. For the reasons that gave rise to hesitation, the questionnaire allowed participants to indicate one or more alternatives in the questions addressed. 

**Figure 1:**
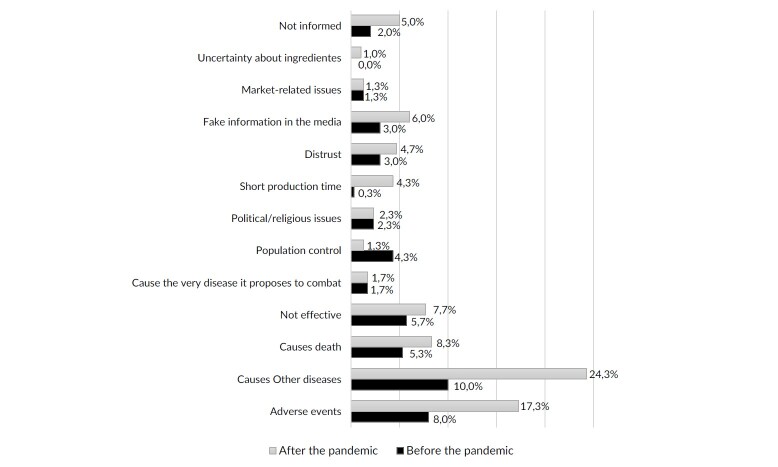
Percent frequency distribution of negative information about vaccines received by healthcare professionals, according to the period of occurrence. Campo Grande, 2022-2023 (n=349)

Responses to open-ended questions were categorized using key terms identified in responses about reasons related to vaccine hesitancy and examples of negative information about vaccines (Table 2 and Figure 1, respectively). The questions asked participants to consider the period before the COVID-19 pandemic and then the period after it. This allowed us to observe any changes in behavior/perception.

**Table 2:** Reasons mentioned by healthcare workers for the occurrence of vaccine hesitancy according to the 3C Conceptual Model. Campo Grande, 2022-2023 (n=213)

3C Model Categories	Vaccine hesitancy (%)
Trust	132 (62.0)
I had a bad experience with a healthcare professional who administered a previous dose	3 (1.4)
I had a bad experience with the vaccine	31 (14.6)
I didn’t think the vaccine was safe and was worried about adverse effects	71 (33.3)
Someone else told me it wasn’t safe	3 (1.4)
Fear of needles	9 (4.2)
Lack of studies proving efficacy and safety	6 (2.8)
I didn’t think the vaccine was effective	9 (4.2)
Complacency	22 (10.3)
Another person told me that she or her children had bad experiences with vaccines.	6 (2.8)
I didn’t think it was necessary	6 (2.8)
Political and religious reasons	2 (0.9)
Other beliefs (alternative medicine)	1 (0.5)
Forgot to take it	7 (3.3)
Convenience	59 (27.7)
I have heard or read negative things about vaccines in the media	15 (7)
I didn’t know where to get good, reliable information about vaccines	8 (3.8)
I couldn’t miss work/classes	11 (5.2)
Lack of knowledge	4 (1.9)
Number of doses	4 (1.9)
Vaccine production time	9 (4.2)
Acute phase disease	5 (2.3)
Unavailability	2 (0.9)
Bureaucracy	1 (0.5)
Total mentions for all options/causes of hesitation	213 (100)

For the analyses, workers were grouped into the following categories: physician; nurse; other higher-level professionals (dentists, pharmacists, social workers, psychologists, physical educators and managers); nursing technicians; community health and disease control agents; and other mid-level professionals (administrative assistants and oral health assistants).

### Study size

Non-probabilistic sampling was adopted for convenience. Each unit was visited once, and workers who agreed to participate in the study were included.

### Statistical methods and bias control

Descriptive statistics were used to characterize the general population studied, which was grouped according to the presence/absence of vaccine hesitancy. The reasons for vaccine hesitancy were based on the 3C model, composed of the three determinants:

- Trust: knowledge and perceptions about the safety and efficacy of vaccines, considering previous experiences of adverse reactions, and credibility given to institutions, services and healthcare professionals who deal with vaccination;
- Convenience: availability, geographical accessibility of services, access and understanding of information; and
- Complacency: low individual perception of the risk of vaccine-preventable diseases (2).

Bivariate analysis was used to assess the statistical association between vaccine hesitancy and demographic, socioeconomic and behavioral profile variables (work processes in primary health care). Categorical variables were analyzed using the chi-square or Fisher’s exact tests and quantitative variables were analyzed using the Mann-Whitney test for comparison of means. 

For the analysis with multiple variables, the binomial logistic regression model was used, with the occurrence of vaccine hesitancy as the outcome. At this stage, only covariates that presented an association with a p-value less than or equal to 0.200 in the bivariate analysis were considered. The stepwise algorithm (with backward and forward directions) and the Akaike information criterion were used to select covariates, control possible confounding factors and define the model with the best fit. The adjusted odds ratio (OR) and 95% confidence interval (95%CI) were used as complementary measures for the interpretation of associations. As an adjustment measure, the Hosmer and Lemeshow test was used. The significance level of 5% (α=0.050) was adopted for all hypothesis tests performed. R software version 4.3.2 was used for the analyses.

## Results

A total of 349 workers from 73 PHC health units agreed to participate in the study (one unit did not participate), and the percentage of vaccine hesitancy was 32.7% (114). Table 1 presents the general characterization of the study population, as well as the frequency distribution of the variables analyzed according to the occurrence of vaccine hesitancy.

**Table 1:** Demographic profile, professional category, perceptions and factors related to vaccination, according to the occurrence of vaccine hesitancy. Campo Grande, 2022-2023 (n=349)

Independent variables	Total (%)	Vaccine hesitancy		p-value
		No (%)	Yes (%)	
**Demographic profile and professional category**				
Sex				0.184
Female	288 (82.5)	189 (80.4)	99 (86.8)	
Male	61 (17.5)	46 (19.6)	15 (13.2)	
**Race/skin color**				0.833
White	139 (39.8)	95 (40.4)	44 (38.6)	
Non-white	210 (60.2)	140 (59.6)	70 (61.4)	
Education				0.851
**High school**	106 (30.4)	71 (30.2)	35 (30.7)	
Higher education	157 (45.0)	104 (44.3)	53 (46.5)	
Specialization	86 (24.6)	60 (25.5)	26 (38.2)	
**Professional category**				0.434
Community health agents and disease control agents	88 (25.2)	59 (25.1)	29 (25.4)	
Other primary and secondary level workers	42 (12.0)	30 (12.8)	12 (10.5)	
Nursing technician	103 (29.5)	67 (28.5)	36 (31.6)	
Nurse	53 (15.2)	39 (16.6)	14 (12.3)	
Physician	22 (6.30)	17 (7.23)	5 (4.39)	
Other higher-level workers	41 (11.7)	23 (9.79)	18 (15.8)	
**Perceptions and factors regarding vaccination**				
**Believes in the protective potential of vaccines**				0.327
Yes	348 (99.7)	235 (100)	113 (99.1)	
No	1 (0.29)	0 (0.00)	1 (0.88)	
**Do you believe that there are reasons that make it difficult for you to be vaccinated on the date recommended by the vaccination schedule**?				<0.001
Yes	27 (7.74)	1 (0.43)	26 (22.8)	
No	322 (92.3)	234 (99.6)	88 (77.2)	
**Do you believe there are reasons why people don’t get vaccinated**?				<0.001
Yes	62 (17.8)	29 (12.3)	33 (28.9)	
No	287 (82.2)	206 (87.7)	81 (71.1)	
**Main means used to obtain information about vaccines**				0.119
Healthcare professionals and the Ministry of Health official sites and similar sites	282 (80.8)	195 (83.0)	87 (76.3)	
Social networks and news sites	47 (13.5)	29 (12.3)	18 (15.8)	
Television	11 (3.15)	8 (3.40)	3 (2.63)	
None (does not seek information about vaccines)	9 (2.58)	3 (1.28)	6 (5.26)	
**Received negative information about vaccines**				0.070
Yes	300 (86.0)	196 (83.4)	104 (91.2)	
No	49 (14.0)	39 (16.6)	10 (8.77)	
**Received institutional guidance to get vaccinated**				0.036
Yes	325 (93.1)	224 (95.3)	101 (88.6)	
No	24 (6.88)	11 (4.68)	13 (11.4)	
**Believes he/she is prepared to guide patients about vaccines**				0.278
Yes	265 (75.9)	183 (77.9)	82 (71.9)	
No	84 (24.1)	52 (22.1)	32 (28.1)	
**Believes that hesitancy or refusal to get vaccinated affects the vaccination coverage of his/her patients**				0.034
Yes	216 (75.8)	151 (79.9)	65 (67.7)	
No	69 (24.2)	38 (20.1)	31 (32.3)	
**Do you know other professionals who are hesitant to accept the vaccines made available by the SUS** (**Brazilian Unified Health System**)?				0.363
Yes	235 (67.3)	154 (65.5)	81 (71.1)	
No	114 (32.7)	81 (34.5)	33 (28.9)	
**Do you know other healthcare professionals who hesitate to recommend vaccines made available by the SUS** (**Brazilian Unified Health System) to patients**?				0.093
Yes	223 (64.5)	142 (61.2)	81 (71.1)	
No	123 (35.5)	90 (38.8)	33 (28.9)	

Considering all participants, the majority were women (82.5%), with an average age of 39.3 years, standard deviation (SD) 9.78 years, non-white race/color (60.2%), with higher education (45%) and an average income of R$ 5,596.00 (SD R$ 4,413.00). The most frequent professional category was nursing technicians (29.5%), followed by community health agents/endemic disease control agents (25.2%) (Table 1). The average length of professional experience in PHC was 9.87 years (SD 7.2 years). 

When asked about the protective potential of vaccines, 99.7% (348) stated that vaccines can protect adults and children from serious diseases (Table 1). Of these, 10.9% (38) began to believe and trust in the protective potential of vaccines only after the start of the COVID-19 pandemic.

When asked if there was any reason why people should not be vaccinated, the majority of workers (82.2%) denied it (Table 1). Among the 17.8% who answered “yes”, the main reasons cited were: “allergy to ingredients” (25.8%), “medical recommendations” (12.9%) or “lack of knowledge” (21.0%). Some workers believed that people should not get vaccinated, because of their “freedom of choice” (9.6%) or due to “lack of confidence” (6.4%) in vaccines. Situations that usually do not represent contraindications for vaccination were also mentioned, such as the presence of “comorbidity” (11.3%) and “side effects from previous vaccinations” (4.9%). The majority (71%) said they held these views even before the start of the COVID-19 pandemic.

Regarding the infodemic of negative information about vaccines, 86% of workers reported having heard negative information (Table 1). Of these, 95.3% chose to get vaccinated despite this information. Contact with this negative information occurred both before and after the start of the COVID-19 pandemic for 36% of those who reported this event. For the remaining 64%, negative information was received exclusively after the start of the pandemic. Among the examples of negative information or misinformation, the belief that “vaccines cause other diseases” stood out, cited both in the pre-pandemic period (10%) and after the start of the pandemic (24.3%). The diseases mentioned in misinformation included: acquired immunodeficiency syndrome (AIDS), acute myocardial infarction, stroke, venous thrombosis, autism, psychiatric disorders and musculoskeletal diseases. Figure 1 details the percentage of negative information reported by healthcare workers, showing that most of the negative information reported occurred more frequently after the start of the pandemic.

When evaluating the main source of information about vaccines, the majority of workers (60.7%) cited the websites and social media profiles of official health entities, followed by consulting other healthcare professionals (20.1%) and accessing social media (13.5%). News websites, television, scientific articles and the lack of consultation or research for information about vaccines were also mentioned, to a lesser extent. Almost all participants (93.1%) reported having received institutional guidance on the importance of vaccines, including information on the vaccination schedule and the requirement for complete vaccination (Table 1).

When asked about knowing other professionals who hesitate to accept vaccines from the vaccination schedule, 67.3% responded positively (Table 1). Among these, 73.6% mentioned that the hesitation occurred after the start of the COVID-19 pandemic, while 26.4% were already hesitant before. Furthermore, 64.5% of participants stated that they knew healthcare workers who hesitated to recommend vaccines to patients (Table 1); of these, 82.9% started to act in this way after the start of the pandemic, while 17.1% already adopted this stance before.

About 75.8% of workers believe that vaccine hesitancy can affect vaccination coverage of the population residing in the territory assigned to their health units. When asked about whether they felt prepared to educate patients in the unit where they work, only 75.9% responded positively (Table 1). 

**Figure 2:**
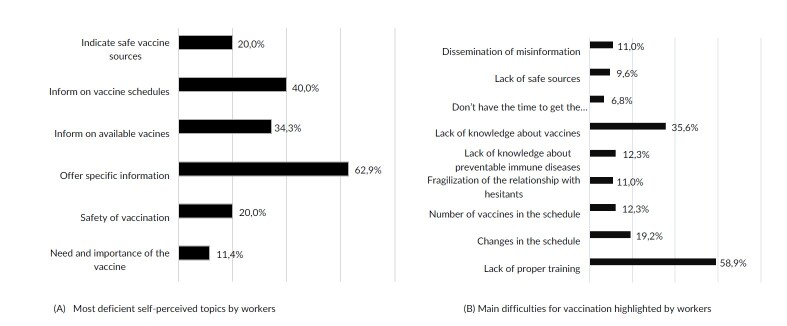
Themes (A) and difficulties (B) reported by healthcare professionals for the transfer of guidance on vaccines to the population served in basic health units. Campo Grande, 2022-2023 (n=349)

The topic with the greatest weakness in knowledge reported was “providing specific information about vaccines” (62.9%), followed by “informing on vaccination schedules” (40%). Regarding operational difficulties or barriers to educating patients, the main factors reported were ‘lack of training’ (58.9%) and ‘lack of knowledge about vaccines’ (36%) (Figure 2).

For 60.5% (211) of workers, residents of the territory where they work do not face difficulties in getting vaccinated. However, 9.2% (32) reported the existence of community leaders who discourage vaccination in the territories assigned to the units where they work. Among these leaders, 71.9% were politicians or religious leaders, 34.4% were health professionals and 9.4% were teachers.

Among hesitant workers, 48.2% (55) reported having refused some vaccine. The demographic profile of those who hesitated is very similar to the profile of the general study population (described in the second paragraph of the Results and in Table 1). Among the vaccines available in the PNI (Brazilian National Immunization Plan), the one that generated the most hesitation was the vaccine against coronavirus-19, cited by 68.4% (78/114) of the hesitant professionals. Among these, 60.3% (47/78) refused this immunobiological. Other vaccines that generated hesitation were: influenza (22/114; 19.3%), double adult vaccine (dT) (4/114; 3.5%), yellow fever (3/114; 2.6%), meningococcal ACWY (2/114; 1.8%), triple viral (1/114; 0.9%), pneumococcal (1/114; 0.9%) and hepatitis B (1/114; 0.9%).

Vaccine hesitancy occurred only after the onset of the COVID-19 pandemic for 64.9% (74) of hesitant workers. Among those who also reported refusal, 80.0% (44) indicated that this occurred during the pandemic period.

The analysis of the determinants of vaccine hesitancy, in which participants could indicate more than one option as the cause of hesitancy, obtained 213 responses and demonstrated that the majority of the factors mentioned belong to the trust category (132/213; 62.0%) of the 3C model, followed by convenience (59/213; 27.7%) and complacency (22/213; 10.3%). Table 2 presents these results, detailing the frequencies observed for each question associated with the determinants of the 3C model. Concern about safety stands out, which was the most cited reason (71) by 108 hesitant people, as well as hesitation resulting from having heard negative information about vaccines.

In the initial stage of the association analysis, only the following variables showed a significant association with vaccine hesitancy: “existence of reasons that make it difficult for professionals to get vaccinated on the date recommended by the vaccination schedule”, “existence of reasons for people not to get vaccinated”, “receiving guidance or recommendations from the institution to get vaccinated” and “believing that hesitancy affects the vaccination coverage of patients treated” (Table 1).

In the multivariate analysis, the following factors remained in the final logistic regression model for workers’ vaccine hesitancy: “sex” and “received negative information about vaccines” as non-significant predictors; “believing that hesitancy affects patients’ vaccination coverage” and “received institutional guidance” as significant predictors associated with a lower chance of hesitancy; and “believing that there are reasons for people not to get vaccinated” as a significant predictor that increases the chance of hesitancy (Table 3). The Hosmer and Lemeshow test was not significant (p-value 0.407), indicating a good fit of the model.

**Table 3:** Adjusted odds ratio (OR) and 95% confidence intervals (95% CI) of vaccine hesitancy in healthcare workers by study variables. Campo Grande, 2022-2023 (n=349)

Covariates	β coefficient (standard error)	p-value	OR (95%CI)
Constant (intercept)	0.351 (0.667)	0.599	1.42 (0.38; 5.28)
Sex (Male)	-0.5145 (0.364)	0.157	0.58 (0.28;0.97)
Do you believe there are reasons why people do not get vaccinated? (Yes)	1.100 (0.324)	0.001	3.01 (1.60; 5.71)
Do you believe that hesitancy or refusal to get vaccinated affects your patients’ vaccination coverage (Yes)	-0.779 (0.301)	0.010	0.46 (0.25; 0.83)
Received institutional guidance to get vaccinated (Yes)	-1.217 (0.497)	0.014	0.30 (0.11; 0.78)
Received negative information about vaccines (Yes)	0.610 (0.443)	0.168	1.84 (0.80; 4.65)

## Discussion

Vaccine hesitancy was listed by the WHO as one of the top ten threats to global health in 2019 (14), highlighting the relevance of this phenomenon to public health. In this study, a high proportion of PHC workers, the level responsible for most vaccination actions in Brazil, reported vaccine hesitancy. This finding is worrying, considering the central role of these professionals in promoting vaccination and expanding vaccination coverage.

The vaccine hesitancy observed among primary health care (PHC) workers was not restricted to the coronavirus-19 vaccine, but also included vaccines already consolidated in the national calendar. Other studies on vaccine hesitancy among healthcare professionals have demonstrated variable percentages, depending on the context and the immunobiological evaluated. In France, for example, 23.1% of the professionals were hesitant about the coronavirus-19 vaccine (15); in Italy, the percentages were 44% among pediatricians and more than 70% for the influenza vaccine (16,17). In Canada, hesitancy towards the coronavirus-19 vaccine was 19.1%, and in the United States, 40.5% (18). In Brazil, before the pandemic, vaccine hesitancy among primary and secondary care health professionals was 25.4% for the influenza vaccine (19).

No single sociodemographic variable appeared to influence the hesitation among the professionals in this study. However, the literature suggests that sociodemographic factors may be significant when combined with others, such as advanced age, difficulty in identifying truthful information about vaccines or low perception of risk (7,18). Hesitation in the younger population may be related to easier access to social networks (15,20). Regarding race/skin color, a study in the United Kingdom showed that the black population was twice as likely to be hesitant, associated with factors such as not being released from work to get the vaccine and little knowledge about vaccines (21).

Doctors and nurses showed less hesitation, with doctors standing out with the lowest percentage (13%). This is relevant, as these professionals, especially in PHC, have influence over patients. Their direct assistance activities can increase public awareness of vaccine-preventable diseases (15,22). Community health agents and disease control agents, professionals working exclusively in PHC, were the most hesitant. Due to their functions, such as home visits and monitoring families, these professionals are often the main health reference for the population (23). Hesitancy among these workers may negatively impact the vaccination decisions of the patients in their care.

Most of the hesitant professionals mentioned the coronavirus-19 vaccine as the most questioned, followed by the influenza vaccine. No studies were found comparing vaccine hesitancy for all vaccines in the national calendar among healthcare professionals. This topic has been explored more after the pandemic, especially in relation to the coronavirus-19 vaccine, and before the pandemic it was limited to the influenza vaccine. Post-pandemic studies are essential to assess the impact of vaccine hesitancy related to other vaccines in the schedule.

The literature points to fear of adverse effects as one of the main predictors of vaccine hesitancy (17,20,24), in addition to concerns about the lack of studies, the effectiveness of vaccines, the perception of low risk and the idea that vaccines would cause diseases such as autism and autoimmunity (16). Lack of trust in health services and in the pharmaceutical industry was also an important factor (18), pointing to worrying gaps in knowledge, particularly among healthcare professionals.

The belief that there are reasons not to vaccinate was statistically significant for vaccine hesitancy among workers in this study. However, there is a need to analyze these reasons so that educational interventions can be implemented. Even in rare cases of contraindications, the guidance should be referral to Special Immunobiological Reference Centers, and not discouragement of vaccination (25), in addition to ensuring that medical contraindications are truly impediments to immunization.

The spread of negative information about vaccines was common among workers, especially after the COVID-19 pandemic. During this period, fake news about vaccines became the most shared on digital media, with the denialism promoted by political leaders amplifying the circulation of this misinformation, which impacted the decision to get vaccinated. Selective vaccine choice movements and the administration of only one dose were some consequences of this false information (27). Even before the pandemic, fake news was already influencing vaccination campaigns, sometimes resulting in delays or impediments to campaigns (28). Healthcare professionals, influenced by this information, can also disseminate it, as occurred with the false association between the human papillomavirus vaccine and promiscuity (11), thrombotic events related to the coronavirus-19 vaccine (15) and the relationship between vaccines and autism (16).

Professionals who received recommendations to get vaccinated showed less hesitation. In countries such as the United Kingdom, Italy and Belarus, the government supported healthcare professionals, encouraging vaccination through educational actions and improving access to vaccines, aiming to increase acceptance (16,21,28). Professionals who were happy with their workplace and had a good relationship with management also demonstrated a lower risk of hesitation (20). Trust in government information about vaccines was essential for their acceptance (20).

Studies indicate that hesitant professionals are less likely to convince hesitant patients and are less likely to answer questions about vaccine safety and efficacy, factors that contribute to patient hesitancy (11,29). These results reinforce the need for a multifaceted approach that combines education, effective communication and institutional policies to support PHC workers. Proposals such as educational events on the efficacy and safety of vaccines, demystifying incorrect information, and highlighting the impact of vaccination on disease transmission, may be useful. Creating channels for professionals to express doubts and concerns, in addition to scheduled time in the units for team discussion about the challenges and benefits of vaccination, are also important measures to involve everyone in the process.

Although most workers perceived that community groups did not face difficulties in getting vaccinated, approximately 40% reported such difficulties, highlighting a significant barrier that deserves attention in the local context.

This study has limitations regarding the number of participants, which is common in studies with primary data, and convenience sampling, which may have compromised the representativeness of the categories of workers. Self-reporting may have caused information bias, as it was not compared with records of vaccine doses received. The global analysis of hesitancy was a methodological option to measure the phenomenon, although it is understood that hesitancy can vary depending on the vaccine, as evidenced in the analyses carried out.

It is important to highlight the novelty of the topic of hesitation among PHC workers, which is still not sufficiently explored in the national context. The results confirm the hesitation by this group, especially in the post-COVID-19 pandemic period. Community agents and other PHC workers are very important in this context and require specific approaches to restore vaccination coverage among the population.

## Data Availability

The database and analysis codes used in the research may be made available after publication upon justified request to the authors.
