# Supplementary material for: Vaccine hesitancy among primary care health workers in Campo Grande, after the COVID-19 pandemic
Source: Epidemiol Serv Saude. 2025 Jun 20;34:e20240481. doi: 10.1590/S2237-96222025v34e20240481.en (PMC12204070; doi:10.1590/S2237-96222025v34e20240481.en)
Supplement: Translation

# Translation: Hesitação vacinal entre trabalhadores de saúde da atenção primária de Campo Grande, após a pandemia de covid-19

**DOI:** 10.1590/S2237-96222025v34e20240481.pt

**Authors:** Danilo dos Santos Conrado, Simony Portela do Carmo Drumond, Ana Isabel do Nascimento, Maria Elizabeth Araújo Ajalla, Cláudia Du Bocage Santos-Pinto, Everton Falcão de Oliveira

**Affiliations:** 1 Universidade Federal de Mato Grosso do Sul, Programa de Pós-Graduação em Doenças Infecciosas e Parasitárias, Campo Grande, MS, Brasil; 2 Universidade Federal de Mato Grosso do Sul, Faculdade de Medicina, Campo Grande, MS, Brasil

**Keywords:** Hesitação Vacinal, Pessoal da Saúde, Atenção Primária à Saúde, Desinformação, Estudos Transversais

## Abstract

**Objetivo:**

Analisar a hesitação vacinal entre trabalhadores da atenção primária à saúde, incluindo os fatores associados a ela.

**Métodos:**

Estudo descritivo transversal realizado entre novembro de 2022 a agosto de 2023 na cidade de Campo Grande, Mato Grosso do Sul, por meio da aplicação de um questionário sobre hesitação vacinal proposto pela Organização Mundial da Saúde. A associação da hesitação vacinal com as variáveis sociodemográficas e percepções dos trabalhadores foi verificada por análise bivariada e com múltiplas variáveis.

**Resultados:**

A hesitação vacinal foi observada em 32,7% dos entrevistados, com maior frequência após o início da pandemia de covid-19 (64,9%). A vacina contra o coronavírus-19 teve a maior frequência hesitação (68,4%). Médicos e enfermeiros foram os menos hesitantes, enquanto os agentes comunitários foram os mais hesitantes. Acreditar na existência de razões para que as pessoas não se vacinem foi associado a hesitação vacinal (*odds ratio* ajustada (OR)) 3,01; intervalo de confiança de 95% (IC95%) (IC95% 1,60; 5,71). Por outro lado, receber orientação institucional para se vacinar (OR 0,30; IC95% 0,11; 0,78) e acreditar que a hesitação afeta a cobertura vacinal da população (OR 0,46; IC95% 0,25; 0,83) foram fatores associados com a baixa frequência de hesitação.

**Conclusão:**

A hesitação vacinal foi frequente entre os profissionais da atenção primária. A pandemia de covid-19 e a infodemia que se seguiu a ela parece ter contribuído para a situação. Destaca-se a necessidade de intervenções voltadas para esses trabalhadores de modo a impactar a cobertura vacinal da população geral.

**Aspectos éticos**

A presente pesquisa respeitou os princípios éticos, obtendo os seguintes dados de aprovação: :

Comitê de ética em pesquisa: Universidade Federal de Mato Grosso do Sul

Número do parecer: 5.200.726

Data de aprovação: 13/1/2022

Certificado de apresentação de apreciação ética: 47947821.0.0000.0021

Registro do consentimento livre e esclarecido: Obtido de todos os participantes antes da coleta.

## Introdução

A vacinação é uma das principais medidas preventivas e de controle de diversas doenças infecciosas, especialmente quando altos percentuais de cobertura são alcançados. Contudo, a adesão à vacinação tem diminuído em todo mundo, resultando na reemergência de doenças anteriormente controladas (1). 

Uma das causas para queda da cobertura vacinal é a hesitação vacinal, definida como o atraso, receio ou mesmo a recusa em se vacinar, independente da disponibilidade das vacinas nos serviços de saúde (2). Para compreender esse fenômeno, em 2011 a Organização Mundial de Saúde (OMS) propôs um modelo conceitual, denominado 3C, onde são elencados fatores que determinam a hesitação vacinal: confiança, complacência e conveniência (2). Em 2018, o modelo foi ampliado para os 5C, incluindo cálculo, que avalia os riscos e benefícios da vacinação, e a responsabilidade coletiva, que se refere à disposição de proteger os outros membros da comunidade pela própria vacinação (3).

Nos últimos anos, a hesitação vacinal tem sido intensificada pela disseminação de falsas notícias, potencializadas pelos conteúdos em mídias digitais (4), contribuindo para o que chamamos de “infodemia”. Esta se refere ao excesso de informações sobre determinado problema em saúde, associadas a momentos históricos de insegurança, como o da recente pandemia de covid-19 (5).

A hesitação pode ser percebida na população geral, mas também profissionais de saúde. Estudos indicam baixa adesão vacinal destes profissionais em cenários pré e pós-pandemia de covid-19, mesmo para as vacinas já estabelecidas na rotina do Programa Nacional de Imunizações (PNI) (6,7). Estima-se que profissionais hesitantes, especialmente aqueles atuantes na atenção primária à saúde (APS), causem impacto na vacinação de seus pacientes e assim, na cobertura populacional (4,8-11).

O trabalho tem como objetivo analisar a hesitação vacinal entre trabalhadores de saúde da APS de Campo Grande, Mato Grosso do Sul, e os fatores que a determinam.

## Métodos

### Delineamento e contexto do estudo

Trata-se de um estudo transversal, realizado em Campo Grande, capital de Mato Grosso do Sul, entre novembro de 2022 e agosto de 2023. À época existiam 74 unidades de atenção primária à saúde no município, sendo 70 localizadas na área urbana e 4 na área rural, com 3.080 profissionais vinculados às mesmas (12). Deste total de participantes, não foi possível quantificar as ocorrências de afastamentos, como licença maternidade e atestado médico, que podem ter afetado o total de profissionais elegíveis ao estudo. Cada unidade foi visitada uma vez pelos pesquisadores de campo, previamente treinados, após contato prévio para o agendamento da visita. Os trabalhadores foram convidados a participar e responder ao questionário no momento da visita ou, posteriormente, por meio da disponibilização do link de acesso ao questionário online na plataforma REDCap.

### Participantes

Foram considerados elegíveis todos os trabalhadores de saúde maiores de 18 anos, que estavam em exercício ativo na APS no momento da realização da pesquisa. 

### Variáveis

A hesitação vacinal foi definida como variável dependente. Dados demográficos, socioeconômicos e comportamentais foram utilizados como variáveis independentes. Foram ainda investigadas razões para hesitação refletidas na percepção dos profissionais em relação às vacinas (elencadas de acordo com modelo teórico 3C), informações negativas sobe vacinas e seus períodos de veiculação, se antes ou pós-pandemia de covid-19. Foram abordadas possíveis barreiras de acesso e influência do ambiente de trabalho na decisão sobre a vacinação.

### Fontes de dados e mensuração

O estudo se baseou em um questionário proposto pela OMS para avaliação da hesitação vacinal (13). Questões sobre a caracterização demográfica e socioeconômica foram incluídas neste questionário. Os participantes foram convidados a respondê-lo e apresentados, inicialmente, à definição de hesitação vacinal da OMS (2): “atraso em aceitar se vacinar ou até mesmo recusar a vacinação, independente da disponibilidade de vacinas no Sistema Único de Saúde (SUS); a hesitação envolve fatores pessoais (como religião, cultura e política), fatores sobre a confiança no sistema de saúde e nas vacinas disponíveis (incluindo medo de efeitos adversos) e na importância da vacinação e conveniência em se vacinar (dificuldade de faltar ao trabalho, não ver necessidade na vacinação etc.)”.

 A partir disso, os próprios participantes responderam se hesitaram ou não. Quando o participante respondeu não ser hesitante na primeira questão “Considerando a definição de hesitação vacinal, você já hesitou em receber alguma vacina?”, mas nas perguntas seguintes respondeu ter recusado alguma vacina ou ter mencionado razões ou motivos para recusar quaisquer vacinas, para fins de análise, ele foi considerado como hesitante. Para os motivos que originaram a hesitação, o questionário permitia que os participantes assinalassem uma ou mais alternativas nas questões abordadas. 

Respostas às questões abertas foram categorizadas, por meio de termos-chave identificados nas respostas sobre os motivos relacionados à hesitação vacinal e os exemplos de informações negativas sobre vacinas (Tabela 2 e Figura 1, respectivamente). As perguntas foram feitas pedindo que os participantes considerassem o período anterior à pandemia de covid-19 e, em seguida, o período posterior à mesma. Isso permitiu observar eventuais mudanças de comportamento/percepção.

**Tabela 2:** Motivos citados pelos trabalhadores de saúde para a ocorrência de hesitação vacinal de acordo com o Modelo Conceitual 3C. Campo Grande, 2022-2023 (n=213)

Categorias do Modelo 3C	Hesitação vacinal (%)
Confiança	132 (62,0)
Tive experiência ruim com profissional de saúde que aplicou dose anterior	3 (1,4)
Tive experiência ruim com a vacina	31 (14,6)
Não achava que a vacina era segura e estava preocupada com os efeitos adversos	71 (33,3)
Outra pessoa me disse que não era segura	3 (1,4)
Medo de agulhas	9 (4,2)
Falta de estudos que comprovem eficácia e segurança	6 (2,8)
Não achei que a vacina fosse eficaz	9 (4,2)
Complacência	22 (10,3)
Outra pessoa me disse que ela ou seus filhos tiveram experiências ruins com vacinas	6 (2,8)
Não pensei que fosse necessário	6 (2,8)
Razões políticas e religiosas	2 (0,9)
Outras crenças (medicina alternativa)	1 (0,5)
Esquecimento	7 (3,3)
Conveniência	59 (27,7)
Ouvi ou li coisas negativas sobre vacinas na mídia	15 (7)
Não sabia onde obter informações boas e confiáveis sobre vacinas	8 (3,8)
Não podia faltar ao trabalho/estudo	11 (5,2)
Falta de conhecimento	4 (1,9)
Número de doses	4 (1,9)
Tempo de produção da vacina	9 (4,2)
Doença em fase aguda	5 (2,3)
Indisponibilidade	2 (0,9)
Burocracia	1 (0,5)
Total de citações para todas as opções/causas de hesitação	213 (100)

**Figura 1:** Distribuição de frequências em percentuais de informações negativas sobre vacinas recebidas pelos profissionais de saúde, segundo o período de ocorrência. Campo Grande, 2022-2023 (n=349)

Para as análises, os trabalhadores foram agrupados nas categorias: médico; enfermeiro; outros profissionais de nível superior (odontólogos, farmacêuticos, assistentes sociais, psicólogos, educador físico e gerentes); técnicos de enfermagem; agentes comunitários de saúde e de combate a endemias; e outros profissionais de nível médio (assistentes administrativos e auxiliar de saúde bucal).

### Tamanho do estudo

Foi adotada amostragem não probabilística por conveniência. Cada unidade foi visitada uma vez e foram incluídos os trabalhadores que aceitaram participar do estudo.

### Métodos estatísticos e controle de viés

A estatística descritiva foi utilizada para caracterização geral da população estudada, que foi agrupada segundo a presença/ausência de hesitação vacinal. Os motivos para hesitação vacinal foram baseados no modelo 3C, composto pelos três determinantes:

- Confiança: conhecimento e percepções sobre segurança e eficácia das vacinas, considera experiências anteriores de reações adversas, e credibilidade dada às instituições, serviços e profissionais de saúde que lidam com a vacinação;
- Conveniência: disponibilidade, acessibilidade geográfica dos serviços, acesso e compreensão das informações; e
- Complacência: baixa percepção individual do risco de doenças imunopreveníveis (2).

A análise bivariada foi utilizada para avaliar a associação estatística entre a hesitação vacinal e as variáveis do perfil demográfico, socioeconômico e comportamentais (processos de trabalho na atenção primária à saúde). As variáveis categóricas foram analisadas com os testes qui-quadrado ou exato de Fisher e variáveis quantitativas com o teste de comparação de médias Mann-Whitney. 

Para a análise com múltiplas variáveis foi empregado o modelo de regressão logística binomial tendo como desfecho a ocorrência de hesitação vacinal. Nesta etapa foram consideradas somente as covariáveis que apresentaram associação com p-valor menor ou igual à 0,200 na análise bivariada. O algoritmo stepwise (com as direções backward e forward) e o critério de informação de Akaike foram utilizados para a seleção das covariáveis, controle de possíveis fatores de confusão e definição do modelo com melhor ajuste. A *odds ratio* (OR) ajustada e intervalo de confiança de 95% (IC95%) foram utilizados como medidas complementares para a interpretação das associações. Como medida de ajuste, foi utilizado o teste de Hosmer e Lemeshow. O nível de significância de 5% (α=0,050) foi adotado para todos os testes de hipótese realizados. O software R versão 4.3.2 foi utilizado para as análises.

## Resultados

Um total de 349 trabalhadores de 73 unidades de saúde da APS aceitaram participar do estudo (em uma unidade não houve adesão), e o percentual de hesitação vacinal foi de 32,7% (114). A Tabela 1 apresenta a caracterização geral da população do estudo, bem como a distribuição de frequências das variáveis analisadas segundo a ocorrência de hesitação vacinal.

**Tabela 1:** Perfil demográfico, categoria profissional, percepções e fatores referentes à vacinação, segundo ocorrência de hesitação vacinal. Campo Grande, 2022-2023 (n=349)

Variáveis independentes	Total (%)	Hesitação vacinal		p-valor
		Não (%)	Sim (%)	
**Perfil demográfico e categoria profissional**				
Sexo				0,184
Mulher	288 (82,5)	189 (80,4)	99 (86,8)	
Homem	61 (17,5)	46 (19,6)	15 (13,2)	
**Raça/cor da pele**				0,833
Branco	139 (39,8)	95 (40,4)	44 (38,6)	
Não-branco	210 (60,2)	140 (59,6)	70 (61,4)	
Escolaridade				0,851
Ensino médio	106 (30,4)	71 (30,2)	35 (30,7)	
Ensino superior	157 (45,0)	104 (44,3)	53 (46,5)	
Especialização	86 (24,6)	60 (25,5)	26 (22,8)	
**Categoria profissional**				0,434
Agentes comunitários de saúde e agentes de combate a endemias	88 (25,2)	59 (25,1)	29 (25,4)	
Outros trabalhadores de nível fundamental e médio	42 (12,0)	30 (12,8)	12 (10,5)	
Técnico de enfermagem	103 (29,5)	67 (28,5)	36 (31,6)	
Enfermeiro	53 (15,2)	39 (16,6)	14 (12,3)	
Médico	22 (6,30)	17 (7,23)	5 (4,39)	
Outros trabalhadores de nível superior	41 (11,7)	23 (9,79)	18 (15,8)	
**Percepções e fatores referentes à vacinação**				
**Acredita no potencial de proteção das vacinas**				0,327
Sim	348 (99,7)	235 (100)	113 (99,1)	
Não	1 (0,29)	0 (0,00)	1 (0,88)	
**Acredita que existam motivos que dificultam a sua vacinação na data recomendada pelo esquema vacinal**				<0,001
Sim	27 (7,74)	1 (0,43)	26 (22,8)	
Não	322 (92,3)	234 (99,6)	88 (77,2)	
**Acredita que exista razões para que as pessoas não se vacinem**				<0,001
Sim	62 (17,8)	29 (12,3)	33 (28,9)	
Não	287 (82,2)	206 (87,7)	81 (71,1)	
**Principal meio utilizado para obter informações sobre as vacinas**				0,119
Profissionais de saúde e sites oficiais do Ministério da Saúde e afins	282 (80,8)	195 (83,0)	87 (76,3)	
Redes sociais e sites de notícias	47 (13,5)	29 (12,3)	18 (15,8)	
Televisão	11 (3,15)	8 (3,40)	3 (2,63)	
Nenhum (não busca informações sobre as vacinas)	9 (2,58)	3 (1,28)	6 (5,26)	
**Recebimento de informações negativas sobre as vacinas**				0,070
Sim	300 (86,0)	196 (83,4)	104 (91,2)	
Não	49 (14,0)	39 (16,6)	10 (8,77)	
**Recebimento de orientação institucional para se vacinar**				0,036
Sim	325 (93,1)	224 (95,3)	101 (88,6)	
Não	24 (6,88)	11 (4,68)	13 (11,4)	
**Acredita estar preparado para orientar os pacientes sobre as vacinas**				0,278
Sim	265 (75,9)	183 (77,9)	82 (71,9)	
Não	84 (24,1)	52 (22,1)	32 (28,1)	
**Acredita que a hesitação ou recusa em se vacinar afeta a cobertura vacinal de seus pacientes**				0,034
Sim	216 (75,8)	151 (79,9)	65 (67,7)	
Não	69 (24,2)	38 (20,1)	31 (32,3)	
**Conhece outros profissionais que hesitam em aceitar as vacinas disponíveis no SUS**				0,363
Sim	235 (67,3)	154 (65,5)	81 (71,1)	
Não	114 (32,7)	81 (34,5)	33 (28,9)	
**Conhece outros profissionais que hesitam em recomendar aos pacientes as vacinas disponíveis no SUS**				0,093
Sim	223 (64,5)	142 (61,2)	81 (71,1)	
Não	123 (35,5)	90 (38,8)	33 (28,9)	

Considerando todos os participantes, a maioria era mulher (82,5%), com média de idade de 39,3 anos, desvio padrão (DP) 9,78 anos), de raça/cor não branca (60,2%), com ensino superior (45%) e média de renda de R$ 5.596,00, (DP R$ 4.413,00). A categoria profissional mais frequente foi a de técnicos de enfermagem (29,5%), seguida por agentes comunitários de saúde/agentes de combate a endemias (25,2%) (Tabela 1). A média do tempo de atuação profissional na APS foi de 9,87 anos (DP 7,2 anos). 

Ao serem questionados sobre o potencial de proteção das vacinas, 99,7% (348) afirmaram que as vacinas podem proteger adultos e crianças de doenças graves (Tabela 1). Destes, 10,9% (38) passaram a acreditar e confiar no potencial protetivo das vacinas somente após o início da pandemia de covid-19.

Quando questionados sobre a existência de alguma razão pela qual as pessoas não devam ser vacinadas, a maioria dos trabalhadores (82,2%) negou (Tabela 1). Entre os 17,8% que responderam “sim”, as principais razões citadas foram: “alergia a componentes” (25,8%), “recomendações médicas” (12,9%) ou “falta de conhecimento” (21,0%). Parte dos trabalhadores acreditava que as pessoas não deveriam se vacinar pelo “direito de escolha” (9,6%) ou pela “falta de confiança” (6,4%) nas vacinas. Situações que usualmente não representam contraindicações para vacinação também foram mencionadas, como a presença de “comorbidade” (11,3%) e “efeitos colaterais em vacinações anteriores” (4,9%). A maioria (71%) afirmou ter essas opiniões antes do início da pandemia de covid-19.

Sobre a infodemia de informações negativas sobre as vacinas, 86% dos trabalhadores relataram ter ouvido informações negativas (Tabela 1). Destes, 95,3% optaram por se vacinar mesmo diante dessas informações. O recebimento dessas informações ocorreu tanto antes quanto após o início da pandemia de covid-19 para 36% dos que afirmaram este acontecimento. Para os demais 64%, as informações negativas foram recebidas exclusivamente após o início da pandemia. Entre os exemplos de informações negativas ou desinformações, destacou-se a crença de que “as vacinas causam outras doenças”, citada tanto no período pré-pandemia (10%) quanto no pós-início da pandemia (24,3%). Para essa desinformação, as doenças mencionadas incluíram: síndrome da imunodeficiência adquirida (aids), infarto agudo do miocárdio, acidente vascular cerebral, trombose venosa, autismo, transtornos psiquiátricos e doenças musculoesqueléticas. A Figura 1 detalha o percentual de informações negativas referidas pelos trabalhadores da saúde, evidenciando que a maioria das informações negativas relatadas teve maior ocorrência após o início da pandemia.

Ao avaliar a principal fonte de informação sobre vacinas, a maioria dos trabalhadores (60,7%) citou os sites e perfis em redes sociais dos órgãos de saúde oficiais, seguidos pela consulta a outros profissionais de saúde (20,1%) e pelo acesso às redes sociais (13,5%). Também foram mencionados, em menor percentual, sites de notícias, televisão, artigos científicos e a ausência de consulta ou pesquisa por informações sobre vacinas. Quase todos os participantes (93,1%) relataram ter recebido orientações institucionais sobre a importância das vacinas, incluindo informações sobre o calendário vacinal e a exigência de vacinação completa (Tabela 1).

Quando questionados sobre o conhecimento de outros profissionais que hesitam em aceitar vacinas do calendário vacinal, 67,3% responderam positivamente (Tabela 1). Dentre esses, 73,6% mencionaram que a hesitação ocorreu após o início da pandemia de covid-19, enquanto 26,4% já eram hesitantes anteriormente. Além disso, 64,5% dos participantes afirmaram conhecer trabalhadores da saúde que hesitam em recomendar vacinas aos pacientes (Tabela 1); destes, 82,9% passaram a atuar dessa forma após o início da pandemia, enquanto 17,1% já adotavam essa postura antes.

Cerca de 75,8% dos trabalhadores acreditam que a hesitação vacinal pode afetar a cobertura vacinal da população residente no território adstrito às suas unidades de saúde. Quando questionados sobre se sentirem preparados para orientar os pacientes da unidade onde atuam, somente 75,9% responderam positivamente (Tabela 1). 

A temática de maior fragilidade de conhecimento relatada foi “prover informações específicas sobre vacinas” (62,9%), seguida por “informar esquemas vacinais” (40%). Em relação às dificuldades ou barreiras operacionais para orientar os pacientes, os principais fatores relatados foram ‘falta de treinamento’ (58,9%) e ‘falta de conhecimento sobre vacinas’ (36%) (Figura 2).

**Figura 2:** Temáticas (A) e dificuldades (B) relatadas pelos profissionais para o repasse de orientações sobre as vacinas para a população atendidas nas unidades básicas de saúde. Campo Grande, 2022-2023 (n=349)

Para 60,5% (211) dos trabalhadores, os moradores do território onde atuam não enfrentam dificuldades para se vacinar. No entanto, 9,2% (32) referiram a existência de líderes comunitários que desencorajam a vacinação nos territórios adstritos às unidades onde trabalham. Entre esses líderes, 71,9% eram políticos ou religiosos, 34,4% profissionais de saúde e 9,4% professores.

Entre os trabalhadores hesitantes, 48,2% (55) relataram ter recusado alguma vacina. O perfil demográfico daqueles que hesitaram é muito semelhante ao perfil da população geral do estudo (descrita no segundo parágrafo dos Resultados e na Tabela 1. Entre as vacinas disponíveis no PNI, a que mais gerou hesitação foi a vacina contra o coronavírus-19, citada por 68,4% (78/114) dos profissionais hesitantes. Dentre estes, 60,3% (47/78) recusaram este imunobiológico. Outras vacinas que geraram hesitação foram: influenza (22/114; 19,3%), dupla adulto (4/114; 3,5%), febre amarela (3/114; 2,6%), meningocócica ACWY (2/114; 1,8%), tríplice viral (1/114; 0,9%), pneumocócica (1/114; 0,9%) e hepatite B (1/114; 0,9%).

A hesitação vacinal ocorreu somente após o início da pandemia de covid-19 para 64,9% (74) dos trabalhadores hesitantes. Entre aqueles que também relataram recusa, 80,0% (44) indicaram que este fato ocorreu durante o período pandêmico.

A análise dos determinantes da hesitação vacinal, na qual os participantes poderiam assinalar mais de uma opção como causa da hesitação, obteve 213 respostas e demonstrou que a maioria dos fatores citados pertencem à categoria confiança (132/213; 62,0%) do modelo 3C, seguida por conveniência (59/213; 27,7%) e complacência (22/213; 10,3%). A Tabela 2 apresenta esses resultados, detalhando as frequências observadas para cada pergunta associada aos determinantes do modelo 3C. Destaca-se a preocupação com a segurança, que foi o motivo mais citado (71) pelos 108 hesitantes, bem como a hesitação decorrente do recebimento de informações negativas sobre as vacinas.

Na etapa inicial da análise de associação, somente as seguintes variáveis apresentaram associação significativa com a hesitação vacinal: “existência de motivos que dificultam a vacinação do profissional na data recomendada pelo esquema vacinal”, “existência de razões para que as pessoas não se vacinem”, “recebimento de orientação ou recomendação da instituição para se vacinar” e “acreditar que a hesitação afeta a cobertura vacinal dos pacientes atendidos” (Tabela 1).

Na análise com múltiplas variáveis, os seguintes fatores permaneceram no modelo final da regressão logística para a hesitação vacinal dos trabalhadores: “sexo” e “recebimento de informações negativas sobre as vacinas” como preditores não significativos; “acreditar que a hesitação afeta a cobertura vacinal dos pacientes” e “receber orientação institucional” como preditores significativos associados a uma menor chance de hesitação; e “acreditar na existência de razões para que as pessoas não se vacinem” como preditor significativo que aumenta a chance de hesitação (Tabela 3). O teste Hosmer e Lemeshow foi não significativo (p-valor 0,407), indicando um bom ajuste do modelo.

**Tabela 3:** Razão de chances (*odds ratio*, OR) ajustada e intervalos de confiança de 95% (IC95%) de hesitação vacinal nos trabalhadores de saúde pelas variáveis do estudo. Campo Grande, 2022-2023 (n=349)

Covariáveis	Coeficiente β (erro padrão)	p-valor	OR (IC95%)
Constante (intercepto)	0,351 (0,667)	0,599	1,42 (0,38; 5,28)
Sexo (Homem)	-0,5145 (0,364)	0,157	0,60 (0,28; 1,19)
Acredita que exista razões para que as pessoas não se vacinem (Sim)	1,100 (0,324)	0,001	3,01 (1,60; 5,71)
Acredita que a hesitação ou recusa em se vacinar afeta a cobertura vacinal de seus pacientes (Sim)	-0,779 (0,301)	0,010	0,46 (0,25; 0,83)
Recebimento de orientação institucional para se vacinar (Sim)	-1,217 (0,497)	0,014	0.30 (0,11; 0,78)
Recebimento de informações negativas sobre as vacinas (Sim)	0,610 (0,443)	0,168	1,84 (0,80; 4,65)

## Discussão

A hesitação vacinal foi listada pela OMS como uma das dez principais ameaças à saúde global em 2019 (14), evidenciando a relevância desse fenômeno para a saúde pública. Neste estudo, uma alta proporção de trabalhadores da APS, nível responsável pela maior parte das ações de vacinação no Brasil, relatou hesitação vacinal. Este achado é preocupante, considerando o papel central desses profissionais na promoção da vacinação e na ampliação das coberturas vacinais.

A hesitação vacinal observada entre os trabalhadores da atenção primária à saúde (APS) não se restringiu à vacina contra o coronavírus-19, abrangendo também imunizantes já consolidados no calendário nacional. Outros estudos sobre hesitação vacinal entre profissionais de saúde demonstraram percentuais variáveis dependendo do contexto e do imunobiológico avaliado. Por exemplo, na França, 23,1% dos profissionais hesitaram em relação à vacina contra o coronavírus-19 (15); na Itália, os percentuais foram de 40% entre pediatras e mais de 70% para a vacina contra influenza (16,17). No Canadá, a hesitação para a vacina contra o coronavírus-19 foi de 19,1%, e nos Estados Unidos foi de 40,5% (18). No Brasil, antes da pandemia, a hesitação vacinal entre profissionais de saúde da atenção primária e secundária foi de 25,4% para a vacina contra influenza (19).

Nenhuma variável sociodemográfica isoladamente pareceu influenciar a hesitação dos profissionais do estudo. No entanto, a literatura sugere que fatores sociodemográficos podem ser significativos quando combinados com outros, como idade avançada, dificuldade em identificar informações verídicas sobre vacinas ou baixa percepção de risco (7,18). A hesitação na população mais jovem pode estar relacionada ao acesso facilitado às redes sociais (15,20). Em relação à raça/cor da pele, estudo no Reino Unido mostrou que a população negra teve o dobro de chances de hesitação, associada a fatores como a não liberação para atividades laborais e pouco conhecimento sobre vacinas (21).

Os médicos e enfermeiros apresentaram menor hesitação, com destaque para os médicos, que tiveram o menor percentual (13%). Isso é relevante, pois esses profissionais, especialmente na APS, têm influência sobre os pacientes. Suas atividades assistenciais diretas podem aumentar a conscientização da população sobre doenças imunopreveníveis (15,22). Os agentes comunitários de saúde e agentes de combate a endemias, profissionais exclusivos da APS, foram os mais hesitantes. Devido às suas funções, como visitas domiciliares e acompanhamento das famílias, esses profissionais são frequentemente a principal referência de saúde para a população (23). A hesitação entre esses trabalhadores pode impactar negativamente as decisões de vacinação dos usuários sob seus cuidados.

A maioria dos profissionais hesitantes referiu-se à vacina contra o coronavírus-19 como a mais questionada, seguida pela vacina contra influenza. Não foram encontrados estudos comparando a hesitação vacinal entre todas as vacinas do calendário nacional entre profissionais de saúde. Esse tema tem sido mais explorado após a pandemia, especialmente em relação à vacina contra o coronavírus-19, e antes da pandemia, limitava-se à vacina contra influenza. Estudos pós-pandemia são essenciais para avaliar o impacto da hesitação vacinal nas outras vacinas do calendário.

A literatura aponta o medo de efeitos adversos como um dos principais preditores da hesitação vacinal (17,20,24), além de preocupações com a falta de estudos, a eficácia das vacinas, a percepção de baixo risco e a ideia de que vacinas causariam doenças como autismo e autoimunidade (16). A falta de confiança nos serviços de saúde e na indústria farmacêutica também foi um fator importante (18), apontando para preocupantes lacunas no conhecimento, principalmente entre profissionais de saúde.

A crença de que existem razões para não vacinar foi estatisticamente significativa para a hesitação vacinal dos trabalhadores deste estudo. No entanto, é necessário analisar essas razões para que intervenções educativas possam ser implementadas. Mesmo nos raros casos de contraindicações, a orientação deve ser o encaminhamento para os Centros de Referência de Imunobiológicos Especiais, e não o desencorajamento à vacinação (25), além de garantir que as contraindicações médicas sejam realmente impeditivas para a imunização.

A disseminação de informações negativas sobre vacinas foi comum entre os trabalhadores, especialmente após a pandemia de covid-19. Durante esse período, as notícias falsas sobre vacinas tornaram-se as mais compartilhadas em mídias digitais, com o negacionismo promovido por líderes políticos amplificando a circulação dessas informações, o que impactou a decisão de se vacinar. Movimentos de escolha seletiva da vacina e a administração de apenas uma dose foram algumas consequências dessas falsas informações (27). Mesmo antes da pandemia, notícias falsas já influenciavam as campanhas de vacinação, resultando, por vezes, em atrasos ou impedimentos nas campanhas (28). Profissionais de saúde, influenciados por essas informações, podem também ser disseminadores delas, como ocorreu com a falsa associação entre a vacina contra o papilomavírus humano e a promiscuidade (11), eventos trombóticos relacionados à vacina contra coronavírus-19 (15) e a relação entre vacinas e autismo (16).

Profissionais que receberam recomendação para se vacinar apresentaram menor hesitação. Em países como Reino Unido, Itália e Bielorrússia, o governo apoiou os profissionais de saúde, incentivando a vacinação por meio de ações educativas e melhoria no acesso às vacinas, visando aumentar a aceitação (16,21,28). Profissionais satisfeitos com o local de trabalho e com boa relação com a gestão também demonstraram menor risco de hesitação (20). A confiança nas informações governamentais sobre vacinas foi essencial para sua aceitação (20).

Estudos indicam que profissionais hesitantes têm menor probabilidade de convencer pacientes hesitantes e são menos propensos a responder dúvidas sobre a segurança e eficácia das vacinas, fatores que contribuem para a hesitação dos pacientes (11,29). Esses resultados reforçam a necessidade de uma abordagem multifacetada que combine educação, comunicação eficaz e políticas institucionais de suporte aos trabalhadores da APS. Propostas como eventos educativos sobre a eficácia e segurança das vacinas, desmistificando informações incorretas, e destacando o impacto da vacinação na transmissão de doenças, podem ser úteis. A criação de canais para que os profissionais expressem dúvidas e receios, além de momentos nas unidades para discussão entre as equipes sobre os desafios e benefícios da vacinação, também são importantes para envolver todos no processo.

Embora a maioria dos trabalhadores tenha percebido que os grupos da comunidade não enfrentavam dificuldades para se vacinar, aproximadamente 40% relataram essas dificuldades, evidenciando uma barreira significativa que merece atenção no contexto local.

Este estudo apresenta limitações em relação ao número de participantes, comum em estudos com dados primários, e à amostragem por conveniência, que pode ter comprometido a representatividade das categorias de trabalhadores. O autorrelato pode ter causado viés de informação, pois não foi confrontado com registros das doses vacinais recebidas. A análise global da hesitação foi uma opção metodológica para dimensionar o fenômeno, embora se entenda que a hesitação pode variar conforme o imunizante, como evidenciado nas análises realizadas.

É importante ressaltar o ineditismo do tema da hesitação entre trabalhadores da APS, ainda pouco explorado no contexto nacional. Os resultados confirmam a hesitação desse grupo, com destaque para o período pós-pandemia de covid-19. Agentes comunitários e outros trabalhadores da APS têm grande importância nesse contexto e demandam abordagens específicas para resgatar as coberturas vacinais entre a população.

## Data Availability

O banco de dados e os códigos de análise utilizados na pesquisa poderão ser disponibilizados após a publicação sob demanda aos autores mediante solicitação justificada.
